# The Advantages and Accidents of Artificial Anæsthesia

**Published:** 1879-02

**Authors:** 


*The Advantages and Accidents of Artificial Anœsthesia.* By Laurence Turnbull, M. D.—Publishers: Lindsay & Blackiston, Philadelphia, 1878.

This is an interesting and comprehensive Manual of Anæsthetic Agents, treating of their relative risk, tests of purity, treatment of asphyxia, spasm of the glottis, syncope. Dr. Bonwill's method for diminishing sensibility by rapid respiration is also noticed, together with the methods of local anæsthesia proposed by Dr. Benj. W. Richardson and others, in the form of ether spray, obtunding mixtures, etc. The medico-legal relations of anæsthetics in regard to the important question whether chloroform can be administered for improper purposes, are duly considered, the general conclusion arrived at being that chloroform cannot be used successfully for felonious purposes, and that a person in the anæsthetic state is not a competent witness. A number of cases are related all going to prove the correctness of the views advanced.

The publication of this manual supplies a want which has existed for some time, and the author has succeeded in furnishing all the information which experience and study have up to this time brought forth.

The dental practitioner will find this manual an important and valuable addition to his professional literature.

